# Artificial Intelligence-Based Automated Analysis for Pleural Effusion Detection on Thoracic Ultrasound: A Systematic Review

**DOI:** 10.3390/diagnostics16010147

**Published:** 2026-01-02

**Authors:** Guido Marchi, Luciano Gabbrielli, Marco Gherardi, Massimiliano Serradori, Francesco Baglivo, Salvatore Claudio Fanni, Jacopo Cefalo, Carmine Salerni, Giacomo Guglielmi, Francesco Pistelli, Laura Carrozzi, Michele Mondoni

**Affiliations:** 1 Pulmonology Unit, Cardiothoracic and Vascular Department, University Hospital of Pisa, Via Paradisa 2, 56124 Pisa, Italy; 2 Italian Society of Artificial Intelligence in Medicine (SIIAM, Società Italiana Intelligenza Artificiale in Medicina), 00165 Rome, Italy; 3 Department of Translational Research and New Technologies in Medicine and Surgery, University of Pisa, 56126 Pisa, Italy; 4 Academic Radiology, Department of Translational Research, University of Pisa, 56126 Pisa, Italy; 5 Respiratory Unit, ASST Santi Paolo e Carlo, Department of Health Sciences, Università degli Studi di Milano, 20122 Milan, Italy; 6 Department of Surgical, Medical and Molecular Pathology and Critical Care Medicine, University of Pisa, 56126 Pisa, Italy

**Keywords:** artificial intelligence (AI), machine learning (ML), deep learning (DL), thoracic ultrasound (TUS), pleural effusion (PE), imaging

## Abstract

**Background**: Pleural effusion (PE) is a common condition where accurate detection is essential for management. Thoracic ultrasound (TUS) is the first-line modality owing to safety, portability, and high sensitivity, but accuracy is operator-dependent. Artificial intelligence (AI)-based automated analysis has been explored as an adjunct, with early evidence suggesting potential to reduce variability and standardise interpretation. This review evaluates the diagnostic accuracy of AI-assisted TUS for PE detection. **Methods**: This review was registered with PROSPERO (CRD420251128416) and followed PRISMA guidelines. MEDLINE, Scopus, Google Scholar, IEEE Xplore, Cochrane CENTRAL, and ClinicalTrials.gov were searched through 20 August 2025 for studies assessing AI-based TUS analysis for PE. Eligible studies required recognised reference standards (expert interpretation or chest CT). Risk of bias was assessed with QUADAS-2, and certainty with GRADE. Owing to heterogeneity, structured narrative synthesis was performed instead of meta-analysis. **Results**: Five studies (7565 patients) published between 2021–2025 were included. All used convolutional neural networks with varied architectures (ResNet, EfficientNet, U-net). Sensitivity ranged 70.6–100%, specificity 67–100%, and AUC 0.77–0.99. Performance was reduced for small, trace, or complex effusions and in critically ill patients. External validation showed attenuation compared with internal testing. All studies had high risk of bias in patient selection and index test conduct, reflecting retrospective designs and inadequate dataset separation. **Conclusions**: AI-assisted TUS shows promising diagnostic performance for PE detection in curated datasets; however, evidence is inconsistent and limited by key methodological weaknesses. Overall certainty is low-to-moderate, constrained by retrospective designs, limited dataset separation, and scarce external validation. Current evidence is insufficient to support routine clinical use. Robust prospective multicentre studies with rigorous independent validation and evaluation of clinically meaningful outcomes are essential before clinical implementation can be considered.

## 1. Introduction

Pleural diseases include a broad spectrum of pathological conditions, often presenting with pleural effusion (PE), and are classified as neoplastic—such as mesothelioma and metastatic lesions—or non-neoplastic, including infections, fibrosis, plaques, and pneumothorax [1,2,3,4]. Common symptoms include dyspnoea, chest pain, and cough, although early stages may be asymptomatic [5,6,7,8]. These disorders are associated with significant morbidity, mortality, and reduced quality of life, representing a frequent cause of hospital admission worldwide, including over 350,000 in the United States annually, with rising global incidence and healthcare costs [9,10,11,12].

PE is among the most frequent thoracic conditions, arising from diverse disorders, including heart failure, infections, malignancies, autoimmune, and inflammatory diseases [4,13,14]. Beyond etiological diagnosis, its presence often indicates disease progression, therapeutic failure, and/or the need for urgent intervention [3,15]. Accurate and timely detection is therefore critical for patient management and outcomes [6,7,16].

Traditional evaluation relies on chest radiography and computed tomography [12,17,18,19,20]. Chest X-rays are widely accessible and inexpensive but with a limited accuracy in detecting small or loculated effusions and differentiating pleural thickening [21]. Chest CT scan provides superior anatomical detail but is costly, it involves greater radiation exposure, and it is often impractical for bedside assessment in critically ill patients [17,18].

Thoracic ultrasound (TUS) has transformed the diagnostic evaluation of pleural disorders. Beyond its established role in guiding safe procedures, it enables bedside assessment of pleural pathology and is increasingly recognised as a field of active research with both clinical and academic significance [22,23,24,25,26,27,28]. It offers real-time imaging, portability, absence of radiation, procedural guidance, and cost-effectiveness, with sensitivity and specificity for PE frequently exceeding 95% [29]. TUS is widely applicable in emergency, intensive care, outpatient, and point-of-care settings [13,30,31,32,33,34,35,36,37].

However, it remains operator-dependent, with accuracy influenced by training, protocol adherence, anatomical familiarity, and patient factors such as obesity or subcutaneous emphysema [38]. Inter-observer variability and technical limitations can reduce diagnostic consistency, highlighting the need for standardised training and supportive technologies [39].

Artificial intelligence (AI) has introduced transformative potential across medicine, enabling data-driven decision support, predictive analytics, and workflow optimisation [40,41,42,43,44,45]. In respiratory medicine, AI has been increasingly explored across diverse domains, including the assessment of pulmonary function, disease phenotyping, and patient risk stratification [46,47,48,49,50,51,52,53]. In imaging, machine learning (ML) and deep learning (DL)—including convolutional neural networks (CNNs)—have enhanced automated recognition, quantification, and pattern detection across organ systems, often matching or exceeding expert human performance [54,55,56,57]. In TUS, AI has been investigated for applications such as automated B-line quantification, pneumonia detection, and pneumothorax identification, showing preliminary evidence of reproducible accuracy and potential to reduce operator dependency [58,59,60,61,62,63,64,65,66]. AI-assisted analysis in PE could have the potential to enhance diagnostic confidence, reduce cognitive burden, expedite interpretation, support training, and facilitate triage in resource-limited settings [57]. It may also contribute to reduced reliance on confirmatory imaging, more efficient workflows, and potential cost savings. Nevertheless, the current evidence remains fragmented, with considerable heterogeneity, limited external validation, and persisting gaps regarding performance in complex cases, integration into clinical practice, and overall cost-effectiveness [67,68]. In addition, beyond diagnostic performance, the interpretability of AI models remains a critical barrier to clinical adoption. Current explainability approaches in AI-assisted PE detection are heterogeneous and often superficial, limiting clinician trust and regulatory readiness [69,70,71,72,73,74].

Given the clinical significance of PE and the transformative potential of AI-assisted TUS, a rigorous synthesis of the existing evidence is warranted. While we recently published a narrative review providing a broad educational overview of advanced imaging and AI across the entire spectrum of pleural diseases and multiple imaging modalities [56], the present work represents a fundamentally different contribution in scope, methodology, and clinical applicability. This systematic review addresses a single, focused clinical question—the diagnostic accuracy of AI-assisted thoracic ultrasound for pleural effusion detection—through rigorous PRISMA-compliant methodology with prospective protocol registration, standardized quality assessment, and certainty of evidence evaluation (detailed in Section 2). Unlike the narrative format, which served as an educational introduction without systematic evidence synthesis, this review systematically aggregates diagnostic performance data across studies, critically appraises methodological quality and generalizability, evaluates explainability implementation, and provides actionable guidance on current clinical readiness and future validation requirements. By systematically examining model architectures, validation strategies, and reported diagnostic performance, this work—representing to our knowledge the first systematic review specifically evaluating this AI application—aims to delineate the current state of evidence, highlight methodological strengths and limitations, and provide guidance for future research and potential clinical integration.

## 2. Methods

### 2.1. Review Design

This systematic review was registered with PROSPERO (CRD420251128416) and conducted in accordance with the Preferred Reporting Items for Systematic Reviews and Meta-Analyses (PRISMA) guidelines [75,76]. The study aimed to evaluate the diagnostic accuracy of AI-based automated analysis applied to TUS for the detection of PE.

Evidence regarding AI model performance was systematically extracted, and methodological quality and certainty of findings were critically appraised. Heterogeneity was assessed across four predefined domains: (1) clinical, including differences in prevalence and patient populations; (2) methodological, encompassing study design (retrospective versus prospective) and validation strategies (cross-validation versus external validation); (3) AI architecture, considering the diversity of model families, input formats, and output types; and (4) technical, addressing variability in data preprocessing and augmentation pipelines.

Given the fundamental differences in prediction tasks [77], model architectures, and input/output formats across studies, quantitative meta-analysis was deemed inappropriate in accordance with Cochrane guidance.

Consequently, a structured narrative synthesis, supported by tabular summaries, was used to comprehensively report diagnostic performance and model characteristics.

The complete PRISMA 2020 checklist is provided as Supplementary Table S1.

### 2.2. Research Question

In patients undergoing TUS for suspected PE (P), how accurately does AI-based automated analysis of the scan (I) detect PE compared with expert interpretation and/or chest CT scan (C), as measured by sensitivity, specificity, and AUC (O)?

### 2.3. Eligibility Criteria

#### 2.3.1. Population

Studies enrolling patients of any age or sex who underwent TUS for suspected PE were eligible. Eligible settings included emergency departments, intensive care units, respiratory or general medicine wards, and outpatient clinics. Only studies written in English were considered, without geographic or socioeconomic restrictions. Excluded were animal or in vitro studies, case reports and studies addressing pleuropulmonary conditions other than PE without separable effusion data.

#### 2.3.2. Index Test

Eligible studies evaluated AI-based automated analysis techniques applied to TUS images or video clips for PE detection. Included AI models comprised DL approaches, CNNs, ensemble methods, or other algorithmic frameworks. Both fully and semi-automated AI systems intended to support or replace human interpretation were considered. Excluded were studies relying solely on manual interpretation, AI applied to other imaging modalities (CT, X-ray, MRI), or studies exclusively focused on other pleuropulmonary conditions without separable PE data.

#### 2.3.3. Reference Standard

Studies were required to include a recognized reference standard for PE diagnosis, including radiologist or expert clinician interpretation, or clinically confirmed diagnosis supported by imaging techniques (chest CT scan or chest X-Ray). Studies without a defined reference standard, comparisons with non-imaging modalities not directly related to PE, or comparisons solely between AI models without a human or standard reference comparator were excluded.

### 2.4. Outcomes

Primary outcomes: Sensitivity, specificity, positive predictive value (PPV), negative predictive value (NPV), overall accuracy, and area under the receiver operating characteristic curve (AUC) of AI-based techniques for PE detection.

Secondary outcomes: Comparative diagnostic performance of AI versus human clinicians; subgroup analyses in specific patient populations (e.g., small or complex effusions, critically ill patients).

### 2.5. Eligible Studies

Eligible study designs encompassed prospective and retrospective cohort studies, diagnostic accuracy studies, cross-sectional investigations, and randomized controlled trials evaluating AI-assisted diagnostic strategies, conducted in either single-center or multicenter settings. Excluded were case reports, small case series (<10 patients), reviews, editorials, commentaries, and conference abstracts lacking complete diagnostic data.

### 2.6. Information Sources and Search Strategy

A systematic literature search was conducted in collaboration with a professional medical librarian. The following databases and sources were systematically searched: MEDLINE (via PubMed), Scopus, Google Scholar, IEEE Xplore, the Cochrane CENTRAL Register of Controlled Trials, and ClinicalTrials.gov. EMBASE was not accessed due to institutional subscription limitations; however, this limitation was mitigated through comprehensive searches of the multiple other relevant databases mentioned above, thereby ensuring broad literature coverage. Searches were performed without date restrictions and were limited to publications in English. All databases and sources were searched from inception through 20 August 2025, with the final search executed on that date.

Search strategies were developed around three core concepts: (i) thoracic ultrasound, (ii) pleural effusion, and (iii) artificial intelligence or automated image analysis. For MEDLINE (via PubMed), controlled vocabulary terms (MeSH) were combined with free-text keywords using the Boolean operator OR. For the remaining sources—keyword-based searches were performed due to the absence of formal controlled vocabulary. The three core concepts were linked using AND across all databases and sources. The complete search strings are provided in the Supplementary Material (Supplementary Material—Full search strategy).

### 2.7. Study Selection and Screening

After duplicates were automatically removed, both reviewers independently screened titles and abstracts using Rayyan.ai software (Rayyan Systems Inc., Cambridge, MA, USA) [78]. Full texts of potentially eligible studies were obtained and assessed independently by two reviewers. Discrepancies were resolved by discussion or consultation with a third reviewer. Standardized screening forms ensured consistency and transparency.

### 2.8. Data Extraction

Data were extracted independently by at least two reviewers using pre-specified forms, with disagreements resolved through discussion or adjudication by a third reviewer. Extracted information included the following:

- Study characteristics: author, year, country, study design (prospective/retrospective), clinical setting, and single- versus multicenter status.
- Patient characteristics: demographics, clinical features, prevalence and characteristics of pleural effusion (size, complexity), and relevant subgroups (e.g., ICU patients).
- Ultrasound technical specifications: probe type, ultrasound system, and acquisition protocols.
- AI system details: model architecture, input data type (still images, frames, or video), level of automation, pre-processing and data augmentation steps, multi-model or multi-label configurations and explainability and interpretability methods.
- Reference standard applied.
- Diagnostic performance metrics: sensitivity, specificity, PPV, NPV, overall accuracy, and AUC.
- Subgroup or secondary analyses.

Individual participant data (IPD) were not sought; extraction relied solely on information reported in the published studies. Missing or unclear information was addressed by contacting study authors when feasible; otherwise, affected data were excluded.

### 2.9. Risk of Bias and Quality Assessment

Risk of bias was evaluated using QUADAS-2, covering patient selection, index test, reference standard, and flow/timing [79]. Assessments were conducted independently by two reviewers. Potential bias due to missing or unpublished results was considered and discussed qualitatively, acknowledging that selective reporting cannot be fully excluded given the small number of included studies.

### 2.10. Certainty of Evidence

The certainty of the synthesized evidence was evaluated in accordance with the GRADE framework [80]. This assessment systematically considered potential limitations across five domains: risk of bias, consistency of results, directness of evidence, precision of effect estimates, and the likelihood of publication bias. Outcomes were subsequently categorised as high, moderate, low, or very low certainty, providing a transparent appraisal of confidence in the reported diagnostic performance of AI-assisted TUS for PE detection.

### 2.11. Data Synthesis

Due to clinical and methodological heterogeneity—including differences in AI models, input data types, reference standards, and reported metrics—formal statistical pooling was not performed. A structured narrative synthesis integrated with four tables was employed to describe diagnostic performance, compare model characteristics, and identify knowledge gaps.

## 3. Results

**Figure 1:**
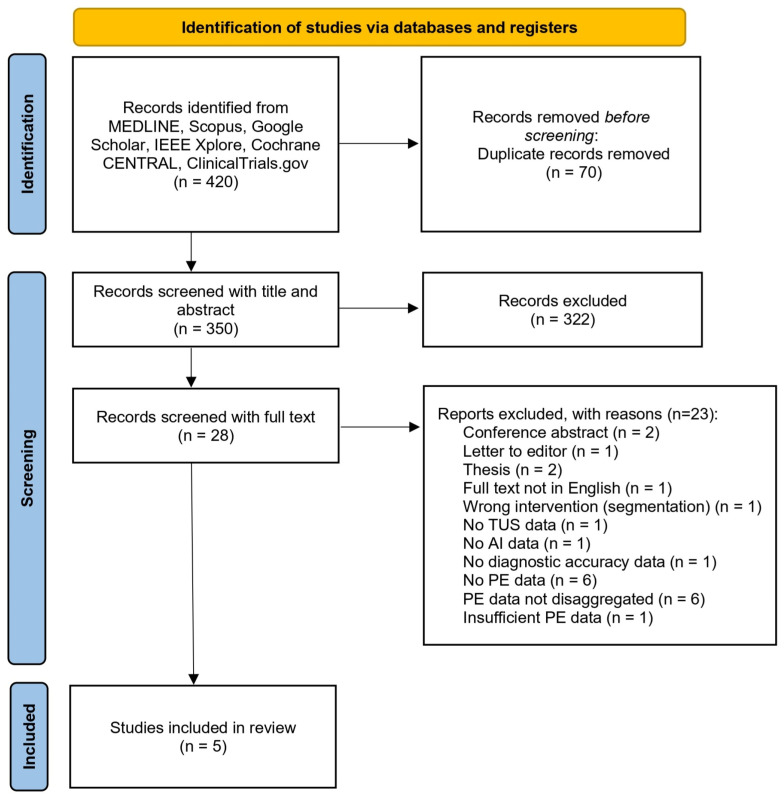
PRISMA flow diagram illustrating the systematic screening, eligibility assessment, and inclusion of studies in the revie.

After completion of screening, five studies were identified which met the eligibility criteria, as illustrated in the PRISMA flow diagram (Figure 1).

The included studies demonstrated considerable heterogeneity across several methodological and clinical domains. To address this, the subsequent subsections present a structured synthesis covering study characteristics and settings, ultrasound technical specifications and acquisition protocols, AI system architecture and data processing, validation strategies and dataset partitioning, diagnostic performance metrics, specialised model configurations and clinical applications, performance stratified by effusion characteristics, and statistical significance with comparative analyses. These findings are further synthesised in three summary tables: Table 1 (Study and testing dataset characteristics), Table 2 (Software characteristics), and Table 3 (Diagnostic accuracy measures).

### 3.1. Study Characteristics and Settings

The included studies were published between 2021 and 2025, with all appearing after 2020 [81,82,83,84,85]. Four adopted a retrospective design, while one was prospective. Across all investigations, interpretation of TUS images by expert clinicians or radiologists served as the reference standard; in addition, one study [84] incorporated chest CT to further validate observer performance. Collectively, the studies enrolled 7565 unique patients across diverse clinical contexts. Sample sizes ranged from 70 patients [81] to 3966 [82], with a median cohort size of 848. Two studies were conducted in China [82,83], one in Australia [81], one in Canada [85], and one in the Republic of Korea [52]. Clinical settings varied substantially, spanning emergency departments, intensive care units, inpatient wards, and general ultrasound departments.

Prevalence of PE varied markedly across studies, ranging from 25.1% [82] to 71.4% [82], with a median of 53.6%. This 2.8-fold variation in disease prevalence has critical implications for interpretation of diagnostic accuracy metrics, particularly positive and negative predictive values.

Three investigations focused exclusively on binary detection of PE [81,83,85], whereas two applied multi-class or multi-label classification approaches that included PE alongside other TUS findings [82,84]. Across all studies, patient populations consisted exclusively of adults (age ≥ 18 years). Mean ages ranged from 59.8 ± 14.9 years (Chen et al. [82]) to 65 ± 17 years (Tsai et al. [81]). No studies included pediatric patients (age < 18 years), representing a significant evidence gap given the increasing utilization of point-of-care ultrasound in pediatric emergency medicine. Detailed study and dataset characteristics are provided in Table 1.

### 3.2. Ultrasound Technical Specifications and Acquisition Protocols

All conventional ultrasound probe types were represented across the included studies. Linear probes were employed in two studies, convex probes in three, and phased array probes in three, with two studies evaluating AI algorithms across multiple probe types. Ultrasound systems varied considerably, encompassing equipment from multiple manufacturers and imaging settings differed between measurements, including frequency, gain, depth, and focal zone adjustments (see Table 1).

Regarding acquisition protocols, two studies implemented established standardized ultrasonography protocols. Hong et al. applied the BLUE (Bedside Lung Ultrasound in Emergency) protocol, assessing six anatomical zones, while Chaudhary et al. followed the Blue Protocol, focusing on the costophrenic regions. The remaining studies employed institution-specific scanning approaches, including a six-region lung division [82] and six anatomical zones according to a standardized TUS protocol [81]. Overall, substantial heterogeneity existed in probe selection, ultrasound systems, and image acquisition protocols, reflecting differences in clinical practice, institutional resources, and study objectives.

No study reported implementation of automated image quality assessment (IQA) algorithms; all quality control was performed through manual expert review, with exclusion criteria varying from ‘poor image quality’ (Tsai, Huang) to ‘excessive probe movement’ (Chaudhary) to ‘images obscured by artifacts’ (Hong).

Details regarding ultrasound equipment, probe types, and acquisition protocols are summarised in Table 1.

### 3.3. AI System Architecture and Data Processing

All five studies employed CNNs as their primary classification approach, although the specific architectures varied substantially. Tsai et al. implemented a Regularised Spatial Transformer Network combined with CNN, Huang et al. compared Attention U-net with standard U-net models, Hong et al. utilised EfficientNet-B0 with transfer learning, Chen et al. developed a ResNet-based model incorporating a Split-Attention mechanism, and Chaudhary et al. created Pleff-Net, a bespoke CNN designed specifically for PE detection.

Among included studies, architectural approaches varied substantially between pure classification and hybrid segmentation-classification tasks. Huang et al. uniquely implemented both segmentation and classification capabilities through their Attention U-net architecture, achieving automated delineation of effusion boundaries (Dice coefficient 0.86, range 0.83–0.90) alongside binary detection. All other studies (Tsai, Chen, Hong, Chaudhary) focused exclusively on classification tasks without pixel-level segmentation output. This distinction is clinically relevant: classification models provide binary/categorical predictions suitable for screening and triage, whereas segmentation models enable quantitative volumetric assessment and procedural guidance for thoracentesis.

Input data formats were heterogeneous across studies: three investigations relied exclusively on still images [82,83,84], one study extracted still images from video sequences [81], and one processed full video clips by decomposing them into individual frames [85]. The total number of images ranged from 1440 [83] to 99,209 [81], with Chaudhary et al. processing over 313,000 frames from video clips.

This variation in data format underpins a key methodological distinction in AI model design: whether models are trained on individual frames or on full video sequences. Three studies (Chen, Huang, Hong) employed frame-based approaches, which benefit from simpler annotation and larger datasets but discard temporal information inherent to respiratory dynamics. Video-based models (Tsai, Chaudhary), in contrast, capture effusion persistence and motion patterns, providing insights into dynamic pleural changes, though they require more labor-intensive frame- or clip-level labeling.

Tsai et al. directly compared these approaches, reporting frame accuracy of 92.4% versus 91.1% for video, with the difference not statistically significant (*p* = 0.422). Chaudhary et al. developed three clip-level prediction algorithms from frame outputs, illustrating that video models can be optimized for different clinical priorities, such as maximizing sensitivity or specificity. Overall, frame-based models are efficient and practical for large datasets, whereas video-based approaches offer enhanced interpretability of temporal dynamics, a consideration that may be critical in certain diagnostic scenarios.

Pre-processing workflows also displayed considerable variability, encompassing DICOM decompression, format conversion, resizing, and normalization. Data augmentation was employed in three studies [83,84,85] and included horizontal flipping, contrast adjustments, and geometric distortions to enhance model generalisability. Details of the AI architectures, input data formats, and pre-processing pipelines are summarised in Table 2.

### 3.4. Validation Strategies and Dataset Splitting

The included studies exhibited substantial variability in their dataset partitioning and validation methodologies. Three investigations employed 10-fold cross-validation [81,83,85], while two studies applied conventional train-test splits [82,84]. Notably, Hong et al. implemented both temporal and external validation across multiple institutions, representing the most rigorous and comprehensive validation framework. Training set proportions ranged from 80% [82] to 90% [81,83].

Two studies were particularly distinguished for their validation robustness. Hong et al. evaluated model performance across four separate datasets, including internal, temporal, and two external test sets, thereby providing extensive insight into generalisability. Chaudhary et al. applied patient-level dataset splitting to avoid data leakage between training and testing phases, enhancing the reliability of performance estimates. Further details on dataset sizes, partitioning strategies, and validation approaches are summarised in Table 1.

### 3.5. Diagnostic Performance Metrics

Diagnostic accuracy exhibited notable variability across studies and model configurations. Sensitivity values ranged from 70.6% (Hong et al., External test 2) to 100% (Chen et al.), with the majority of investigations reporting sensitivities above 90%. Specificity similarly varied, ranging from 67.0% (Chaudhary et al., Trauma model) to 100% (Chen et al.), with most studies achieving values exceeding 85%.

Overall accuracy spanned from 79.0% (Chaudhary et al., Trauma model) to 98.2% (Chen et al., multi-class classification), with four out of five studies reporting accuracies above 89%. The AUC was reported in four studies, ranging from 0.77 (Hong et al., External test 2) to 0.998 (Chen et al.), reflecting generally strong discriminatory capability.

Notably, performance heterogeneity was particularly evident in multi-institutional validations. Hong et al. observed a progressive decline in AUC across validation datasets, decreasing from 0.94 in internal and temporal tests to 0.89 in External test 1, and 0.77 in External test 2. This pattern highlights potential overfitting and limited generalisability when applying models across different clinical environments.

Variation in disease prevalence (25–71%) markedly affects predictive values: high-prevalence settings inflate PPV but reduce NPV, while low-prevalence settings do the opposite.

Comprehensive diagnostic performance metrics for all included studies are summarised in Table 3.

### 3.6. Specialised Model Configurations and Clinical Applications

Two studies implemented multiple model variants specifically optimised for distinct clinical scenarios. Chaudhary et al. developed three separate models: a general model achieving 90.3% sensitivity and 89.0% specificity, a large-effusion model with 95.5% sensitivity, and a trauma-focused model yielding 98.0% sensitivity and 67.0% specificity. These results demonstrate the adaptability of AI algorithms to differing clinical requirements and effusion characteristics.

Huang et al. directly compared two architectural approaches, reporting that the Attention U-net consistently outperformed the standard U-net across multiple performance metrics, including specificity, precision, accuracy, and F1-score (*p* < 0.05). Moreover, this study incorporated image segmentation capabilities, achieving Dice coefficients of 0.86 for Attention U-net and 0.82 for standard U-net, highlighting the added value of architectural optimisation in AI-assisted pleural effusion detection.

Relevant model configurations, performance metrics, and segmentation outcomes are summarised in Table 2 and Table 3.

### 3.7. Performance by Effusion Characteristics

Three studies conducted subgroup analyses to evaluate diagnostic performance according to effusion characteristics. Chaudhary et al. demonstrated that sensitivity varied with effusion size, achieving 95.1% for large effusions, 92.2% for small effusions, 79.9% for trace effusions, and 77.0% for complex effusions.

Hong et al. assessed performance across different patient populations and reported diminished accuracy in critically ill ICU patients relative to general hospital populations.

Subgroup performance data stratified by effusion size and patient population are summarised in Table 1 and Table 3.

### 3.8. Statistical Significance and Comparative Analyses

Statistical comparisons between models or methodological approaches were reported in three studies. Huang et al. identified statistically significant differences between Attention U-net and standard U-net architectures across multiple performance metrics, with *p*-values ranging from 0.003 to 0.027, favouring the Attention U-net model. In contrast, Tsai et al. observed no significant difference between video-based and frame-based classification approaches (*p* = 0.422). Hong et al. reported a significant improvement in inter-reader agreement for binary pleural effusion detection when AI assistance was provided, with κ values increasing from 0.73 to 0.83 (*p* = 0.01).

Direct comparisons between AI models and human readers are nearly absent. Only Hong et al. assessed AI-assisted human performance, showing modest improvements, but did not evaluate standalone AI versus expert performance. This limits the understanding of whether AI can match or exceed human accuracy, a key requirement for autonomous clinical deployment. Contributing factors include reference standard circularity, resource-intensive reader studies, and potential publication bias. Evidence from related domains [86], suggests AI often approaches average expert performance but may underperform subspecialists, highlighting the need for rigorous comparative evaluation before clinical implementation.

Relevant statistical analyses and comparative performance metrics are summarised in Table 2 and Table 3.

### 3.9. Explainability and Interpretability: A Critical Gap in Clinical Translation

Explainability implementation in AI-based TUS studies varied considerably, with most studies providing only rudimentary interpretability analyses. Gradient-weighted class activation mapping (Grad-CAM) was the predominant method, applied in three studies to generate heatmaps that highlighted image regions influencing model predictions. Tsai et al., by contrast, employed spatial transformer networks, which offered implicit localization capabilities, yet no formal visualization or assessment of attention patterns was provided. Huang et al. relied exclusively on attention mechanisms embedded in the network architecture, but these were neither visualized nor validated, leaving the internal model reasoning largely opaque.

Among the reviewed studies, Chaudhary et al. delivered the most comprehensive explainability assessment. Their approach combined Grad-CAM heatmaps with temporal confidence plotting across video sequences, allowing for the identification of model focus on clinically relevant structures, including anechoic fluid collections, bony structures responsible for the “spine sign” artifact, and pleural line demarcation. Importantly, this temporal analysis enabled the model to distinguish effusions visible during expiration from those compressed during inspiration, reflecting an understanding of respiratory dynamics that is crucial for accurate interpretation of Pes. Despite this sophistication, the study did not include a clinician assessment to verify whether the highlighted regions aligned with expert visual attention patterns.

Chen et al. implemented Grad-CAM within a multi-class ResNet framework, demonstrating class-specific attention patterns for A-lines, B-lines, consolidation, and PE. While these visualizations provided some insight into model reasoning, their clinical validation was limited to visual inspection, without systematic evaluation of whether the attention patterns corresponded to diagnostically meaningful features. Hong et al. briefly mentioned the use of Grad-CAM, but offered minimal analysis of the resulting activation maps. Huang et al. described the theoretical benefits of their attention mechanism, yet did not visualize attention weights or validate their clinical significance. Tsai et al. provided no explainability assessment beyond the implicit localization potential of the spatial transformer.

Critically, none of the studies conducted formal validation of explanation quality, either through clinician assessment or quantitative evaluation of attention map faithfulness. This represents a substantial limitation for clinical translation, as it precludes confirmation that AI models focus on the correct diagnostic features rather than spurious artifacts. The absence of validated explainability impedes regulatory approval, hinders clinician trust, limits error detection, and diminishes potential educational value. Without robust demonstration that model explanations align with expert reasoning and reliably highlight pathologically relevant structures, these AI models remain “black boxes,” unsuitable for autonomous deployment in clinical practice [87].

Details of the explainability approaches and their implementation across studies are summarised in Table 2.

### 3.10. Risk of Bias

The methodological quality of the included studies was systematically appraised using the QUADAS-2 tool, covering the domains of Patient Selection, Index Test, Reference Standard, and Flow and Timing, as well as overall applicability. All assessments were performed independently by two reviewers, with disagreements resolved by discussion or adjudication by a third reviewer to ensure methodological rigour.

All five studies were at high risk of bias for Patient Selection, reflecting predominantly retrospective designs, non-consecutive enrolment, enriched disease prevalence, and systematic exclusion of technically suboptimal scans, which likely inflated performance estimates by omitting challenging real-world cases.

All studies were also rated high risk for Index Test conduct, primarily due to inadequate patient-level dataset partitioning. Except for Chaudhary et al., who explicitly ensured no patient’s images appeared in both training and testing sets, most studies randomized at the image level, allowing multiple images from the same patient in both sets. This AI-specific data leakage enables memorization of patient-specific features rather than generalizable disease patterns, likely inflating performance estimates.

Reference Standard and Flow and Timing were generally rated low risk; however, reliance on expert interpretation of the same images used for model training introduces incorporation bias that may further inflate concordance measures. The combined impact of these biases is likely to result in overly optimistic sensitivity and specificity relative to performance in unselected clinical populations.

Four studies (Tsai, Chen, Huang, Chaudhary) relied solely on expert LUS interpretation as the reference standard, introducing circularity bias, since the same modality served as both index test and comparator. This may artificially inflate diagnostic accuracy compared to an independent gold standard. Only Hong et al. used same-day chest CT for a subset of cases, providing validation against a modality-independent reference. Circularity bias is particularly concerning when AI is trained on expert labels and evaluated against the same experts, as models inherently replicate both correct interpretations and systematic expert errors.

Methodological work in related diagnostic AI domains (e.g., addressing class imbalance, feature selection, and missing data) underscores the importance of balanced datasets and robust validation to mitigate bias and improve generalisability [88]. Overall applicability was judged low risk, indicating alignment between study populations, index tests, and the clinical question addressed in this review.

These evaluations are summarised in Table 4, providing a structured overview of risk of bias and applicability across the included studies.

### 3.11. Certainty of Evidence (GRADE)

The certainty of evidence regarding the diagnostic accuracy of AI-assisted TUS for PE detection was systematically evaluated using the GRADE framework, focusing on sensitivity, specificity, and overall accuracy.

Risk of Bias: Considering the high risk of bias for patient selection and index test reported in most studies (see Table 4), confidence in the effect estimates is tempered by the predominantly retrospective designs and partially overlapping or non-independent datasets. Reference standards and flow and timing were consistently applied across studies, supporting internal validity.

Inconsistency: Substantial heterogeneity was observed among studies in study design (retrospective vs. prospective), sample size (70–3966 patients), clinical settings (emergency department, intensive care unit, hospital wards, ultrasound departments), imaging protocols, and AI model architectures. This heterogeneity was reflected in the wide range of diagnostic performance metrics reported (sensitivity 70.6–100%, specificity 67–100%, AUC 0.77–0.998).

Indirectness: Populations, index tests, and clinical settings were generally aligned with the review question. However, several models were developed using single-centre cohorts or selected patient subpopulations, which may limit generalisability to broader clinical environments.

Imprecision: Variability in sample sizes and the width of confidence intervals, particularly in multi-institutional external validations, contributed to imprecision of effect estimates.

Publication Bias: Formal statistical assessment was constrained by the small number of included studies. Although selective reporting cannot be entirely excluded, the inclusion of both favourable and less favourable outcomes mitigates, but does not eliminate, this concern.

Overall Certainty: Integrating these domains, the certainty of evidence was rated as moderate for sensitivity and specificity, indicating that AI-assisted TUS generally demonstrates high diagnostic accuracy for pleural effusion detection, while acknowledging that methodological limitations and heterogeneity reduce absolute confidence in the estimates. Certainty regarding broader applicability across diverse clinical settings was considered low-to-moderate.

Overall, while AI-assisted TUS demonstrates generally high diagnostic accuracy for PE detection, methodological limitations and heterogeneity moderate the certainty of the evidence.

## 4. Discussion

### 4.1. Interpretation of Findings

This is, to our knowledge, the first systematic review aimed at studying the accuracy of AI-assisted TUS for the detection of PE.

The evidence from the five included studies (n = 7565) confirms that AI-based automated analysis of TUS can achieve high diagnostic accuracy for PE in curated datasets. Reported sensitivities and specificities were frequently >90% in development and internal testing and reported AUCs ranged approximately 0.77–0.998, demonstrating strong discriminatory capability under favourable conditions. Single-centre development studies produced the most optimistic metrics, while temporal and external validations, most notably Hong et al., revealed performance attenuation on independent datasets. Subgroup analyses consistently show reduced accuracy for trace, small or morphologically complex effusions and for critically ill patients, and specialised model variants illustrate clinically relevant trade-offs between prioritising sensitivity in urgent contexts and preserving specificity in screening or routine care. Collectively these results support the technical feasibility of AI assistance for PE detection using TUS, while emphasising that real-world reliability depends on dataset heterogeneity, robust validation strategies and the intended clinical application.

### 4.2. Study Limitations and Methodological Considerations

The current evidence base for AI-assisted TUS is characterised by substantial methodological heterogeneity, limiting interpretability and generalisability. Included studies varied widely in input data (still images versus video clips), ultrasound equipment and acquisition protocols (standardised BLUE-based versus local approaches), AI architectures and preprocessing pipelines (ResNet variants, EfficientNet, U-Net/Attention U-Net, bespoke CNNs), and validation strategies. Most relied on internal splits, with only a minority implementing external or patient-level validation to mitigate data leakage. Reference standards were inconsistent, predominantly based on expert interpretation of the same ultrasound images used for model training, with limited use of independent modalities such as chest CT, raising concerns about circularity. Operator-dependent acquisition, heterogeneous handling of low-quality scans, and video-level rather than frame-level labelling further bias performance estimates. Conventional quality assessment tools, including QUADAS-2, have recognised limitations when applied to AI studies.

Given these factors, quantitative pooling of diagnostic performance was deemed methodologically inappropriate. Architectural differences alone can produce substantially divergent results, even within identical datasets, as demonstrated in other diagnostic AI domains [77]. The included studies evaluated five architecturally distinct models across heterogeneous datasets with widely varying prevalence (25–71%), clinical settings, and validation designs, making pooled sensitivity and specificity estimates non-comparable. Variation in disease prevalence further limits generalisability, as predictive values are prevalence-dependent even when sensitivity and specificity remain constant. Models trained in high-prevalence populations may underperform in low-prevalence settings, with external validation drops illustrating this effect. Standardised predictive value calculations at clinically relevant prevalence can aid interpretation, but prospective validation at intended clinical prevalence is essential. Consequently, a structured narrative synthesis was adopted to preserve critical contextual and methodological information that meta-analysis would obscure.

Explainability and clinical interpretability remain critical gaps. Few studies implemented even basic visualisations, and none provided comprehensive analyses aligned with clinical reasoning or regulatory requirements. Approaches ranged from detailed temporal analyses to complete absence of interpretability evaluation, reflecting the early stage of explainable AI (XAI) in this field. Effective deployment requires anatomically accurate feature focus, real-time confidence assessment, failure-mode highlighting, and integration into clinical workflows; current evidence shows these remain largely unvalidated.

The evidence base is further limited by retrospective designs, selection bias, small sample sizes, and scarce external validation. QUADAS-2 assessments indicate high risk of bias in patient selection and index test conduct, with idealised test conditions—such as exclusion of poor-quality images, inadequate patient-level data separation, and threshold optimisation—likely inflating sensitivity and specificity. Reported metrics should therefore be interpreted as potentially overestimated. No study reported patient-centred outcomes, safety endpoints, or health-economic analyses, and direct AI versus human comparisons are lacking. Applicability is constrained by equipment and protocol heterogeneity, and the small number of studies combined with predominantly positive findings suggests potential publication bias.

Finally, a major limitation is the absence of pediatric data, as all included studies enrolled adult populations (≥18 years). This gap is clinically relevant given the growing use of point-of-care ultrasound in pediatric settings, age-related anatomical and etiological differences in pleural effusion, and distinct pediatric decision thresholds for intervention. AI models trained solely on adult populations cannot be assumed to generalise to children without dedicated validation.

### 4.3. Implications for Clinical Practice

Given the current evidence, AI-assisted TUS shows potential as a complementary decision-support tool, but its role as a reliable and effective adjunct to comprehensive clinical judgment is not yet fully confirmed and remains to be established. These systems appear promising in democratizing access to high-quality ultrasound interpretation, particularly in emergency or resource-limited settings, and as part of telemedicine or teleconsultation workflows, by supporting less-experienced operators and standardizing interpretation. Implementation requires institution-level validation against local devices, probe types and patient mixes, and must include transparent presentation of model confidence and localisation (for example segmentation overlays) together with clear escalation pathways for equivocal results. Ultrasound differs from other forms of automated imaging in that the process of image acquisition itself directly influences diagnostic outcomes, and imperfect scans are unavoidable, especially in fast-paced or high-pressure clinical settings. Incorporating software solutions that assist operators during acquisition and evaluate scan quality in real time could allow immediate identification and exclusion of substandard images, enhance performance for less-experienced users, and increase the overall reliability of AI-supported interpretations. Integration with conventional radiological pathways and clinical assessment remains essential: AI outputs should always be interpreted in the context of clinical history, physical examination, laboratory tests, and integrated with imaging such as TUS, chest radiography, or CT, as part of a comprehensive, multimodal clinical assessment. Practical deployment further demands governance for responsibility of final interpretation, continuous performance monitoring and procedures for handling inadequate scans; embedding an automated scan-quality check within the pipeline could reduce false reassurance from poor inputs. For specific use cases, models should be tailored to context-specific performance priorities (for example, prioritizing high sensitivity for PE detection in critically ill ICU patients, or emphasizing robustness to operator variability in emergency or remote settings where inexperienced operators perform scans).

### 4.4. Research Gaps and Future Directions

Advancement toward safe and generalisable clinical tools requires prospective, multicentre studies with consecutive enrolment across diverse healthcare settings and device vendors, generating realistic, prevalence-dependent performance estimates. Datasets must incorporate technically challenging cases, such as obesity and subcutaneous emphysema, while dedicated paediatric cohorts are essential due to anatomical differences, distinct disease aetiologies, and varying clinical decision thresholds. AI models intended for broad clinical use should therefore be trained on mixed-age datasets or explicitly developed and validated in paediatric populations before deployment in such settings, ensuring generalisability and equity.

Rigorous external and temporal validation should become standard practice, accompanied by transparent reporting of calibration, decision-curve analyses, and uncertainty intervals. Comparative studies should include direct AI-versus-human evaluation, using identical test sets and gold-standard references to determine non-inferiority or complementary error patterns for human–AI synergy.

Clinically validated explainability frameworks are critical and should combine technical visualisation (e.g., Grad-CAM or attention maps), multi-reader assessment, and real-time guidance to highlight diagnostically relevant features, quantify uncertainty, and support confidence-calibrated predictions. These frameworks should incorporate quantitative evaluation of explanations, including faithfulness scores derived from perturbation analysis, pointing game accuracy against expert annotations, multi-reader Likert-scale assessment of explanation utility, and counterfactual testing to ensure that explanations faithfully reflect model reasoning rather than post hoc rationalization. Such explainability measures are essential not only to enhance interpretability and clinician trust, but also to identify model limitations and error patterns, particularly for less-experienced operators.

Future methodological work should further address operator dependence through integrated scan-quality assessment and real-time acquisition support, and explore emerging model architectures to enhance robustness and generalisability. Cost-effectiveness analyses and pragmatic randomized trials are necessary to evaluate AI’s impact on patient outcomes, procedural safety, and resource utilisation. Finally, human-factors research, post-market surveillance, and adherence to AI-specific reporting and validation standards will be crucial to ensure transparent, reproducible, and safe translation of AI tools into routine clinical practice.

## 5. Conclusions

AI-assisted analysis of TUS for the detection of PE represents a rapidly and exponentially evolving domain with substantial translational potential. Current evidence indicates that these algorithms appear capable of achieving diagnostic performance approaching that of expert operators, thereby enhancing reproducibility and standardisation of sonographic interpretation.

Nevertheless, such tools must always be considered adjunctive rather than substitutive, and cannot replace comprehensive clinical assessment, which remains grounded in detailed history-taking, physical examination, symptom evaluation, laboratory testing, and holistic patient evaluation. These methodologies are inherently complementary and should be integrated with conventional radiological imaging and comprehensive clinical assessment to ensure a multidimensional diagnostic approach.

Their strategic implementation may be particularly beneficial in settings with limited specialist expertise, especially in the emergency departments and intensive care units of rural or remote healthcare facilities, as well as in low- and middle-income countries. Furthermore, emerging evidence suggests potential utility even in tertiary care or high-volume academic centres, where AI-assisted interpretation may support workflow efficiency, rapid decision-making, and standardisation of sonographic evaluation.

In all these contexts, integration with telemedicine platforms or remote expert consultation may further augment diagnostic reliability and clinical decision-making. However, current evidence also highlights a gap between technical AI performance and clinical interpretability; robust explainability frameworks, including clinical validation and real-time interpretability interfaces, will be crucial to ensure trust, transparency, and safe deployment.

Future research should prioritise prospective, multicentre studies with rigorous external validation across heterogeneous populations and healthcare environments, alongside systematic evaluation of clinically meaningful outcomes. With continued methodological refinement, adherence to standardised reporting frameworks, and careful integration into clinical workflows, AI-assisted TUS has the potential to serve as a high-value adjunct to conventional imaging and comprehensive clinical evaluation, optimising diagnostic precision, efficiency, and equity in respiratory care.

Beyond local implementation, its development and uptake may also contribute to improving respiratory health in diverse global settings, where disparities in access and resources remain major challenges.

## Figures and Tables

**Table 1:** Study and testing dataset characteristics: Summary of the included studies’ design, centre type, reference standard, patient numbers, dataset characteristics, ultrasound equipment, and clinical indications. LUS = lung ultrasound, PACS = Picture Archiving and Communication System, MHz = megahertz, *n* = number of images/frames/patients, ICU = intensive care unit, US = ultrasound.

Study	Year	Study Design and Centre	Reference Standard	Patient Number	Original Format + Data Number (n)	Dataset Split (Training/Validation/Testing)	Setting	Ultrasound Machine(s)	Ultrasound Probe(s)	Clinical Indication
Tsai et al. [81]	2021	Retrospective, single-centre	Expert clinician interpretation (iLungScan™ validated by LUS experts)	70	Still images: 99,209 from 623 videos	10-fold cross-validation (90% training, 10% testing)	Hospital (Royal Melbourne Hospital)	SonoSite X-Porte	1–5 MHz phased array transducer (rP19xp)	Image evidence of pleural effusion or normal lungs
Chen et al. [82]	2023	Prospective, single-centre	Two experienced ultrasonographers (>5 years experience)	3966	Still images: 5545 total (1394 pleural effusion)	Simple train-test split (80% training, 20% testing)	Hospital ultrasound department	LOGIQ E9 (GE, Wauwatosa, WI)	Convex probe (2.8–5.0 MHz)	Routine LUS following Blue Protocol
Huang et al. [83]	2024	Retrospective, single-centre	Manual annotation by two experienced sonographers	848	Still images: 1440 (800 positive, 640 negative) expanded to 5760 with augmentation	10-fold cross-validation (90% training, 10% testing)	Hospital (ultrasound departments from PACS)	25 models (Philips IU22, EsaoteMyLabTwice, Philips Epiq7C, GE, others)	Not reported	General ultrasound examinations
Hong et al. [84]	2024	Retrospective, multi-centre	Expert radiologist interpretation; chest CT for observer performance test	1898 total (1645 development + 146 temporal + 54 external 1 + 53 external 2)	Still images: 7580 from 1645 patients	Training: Validation: Internal test = 8:1:1 plus external validation	Multi-centre: Seoul National University Hospital and Chung-Ang University Hospital	LOGIQ E9 (GE), EPIQ 5G/7G (Philips), RS80A (Samsung)	High-resolution linear (7–11 MHz) or convex (3–5 MHz)	Patients undergoing thoracentesis, critically ill ICU patients
Chaudhary et al. [85]	2025	Retrospective, multi-centre	Expert LUS interpretation (fellowship-trained expert and Critical Care physician)	785	Video clips: 1664 total (566 with effusion) deconstructed into 313,109 frames	85% development, 15% holdout with 10-fold cross-validation	Two Canadian tertiary hospitals (emergency department, ICU, hospital ward)	Mindray (n = 348), Sonosite (n = 1298), Philips (n = 14), Esaote (n = 3)	Phased array (n = 1395), Curved linear (n = 158), Linear (n = 2)	Routine LUS following Blue Protocol

**Table 2:** Software characteristics. Details of AI classification software and pipelines used in each study, including algorithm type, input data format, pre-processing steps, training dataset, and software implementation. CNN = convolutional neural network, RGB = red-green-blue, DICOM = Digital Imaging and Communications in Medicine, AG = attention gate, Reg-STN = Regularised Spatial Transformer Network, U-net = U-shaped convolutional network architecture for segmentation, ResNet = residual neural network, Mish = Mish activation function, Focal loss = loss function for class imbalance, PNG = Portable Network Graphics format, AI = artificial intelligence.

Study	Model Type	Classification Software	Additional AI Tools in Pipeline	Classifier Final Format	Training Dataset	AI Algorithm Type	Input Data Format	Pre-Processing Steps	Software Implementation	Explainability Approach and Evaluation
Tsai et al. [81]	Classification	Deep learning CNN with Regularised Spatial Transformer Network (Reg-STN)	Spatial Transformer Network for weakly supervised localisation	Binary (pleural effusion/no pleural effusion)	Same institution, 99,209 images from 623 videos	Deep learning using Reg-STN with CNN	Still images from videos	DICOM decompression, overlay removal, cropping to ultrasound sector	Custom implementation based on Roy et al. architecture	Spatial transformer localization (not visualized); no clinical validation; explanation quality not assessed
Chen et al. [82]	Classification	Deep learning CNN (ResNet-based with Split-Attention)	Split-Attention mechanism, Mish activation function, simplified focal loss	Multi-class (A-line, B-line, pulmonary consolidation, pleural effusion)	4436 images from total 5545	Deep learning CNN with Split-Attention	Still images (DICOM format, single RGB channel)	Standardisation, irrelevant fields removed	Custom model (source code to be published on GitHub)	Grad-CAM visualization; limited visual inspection; explanation quality not assessed
Huang et al. [83]	Segmentation + Classification	Attention U-net and U-net models	Attention gates (AGs) for improved feature extraction	Binary with segmentation	Same institution, 5184 images for training (2592 + 2592 annotated)	Deep learning—Attention U-net and U-net	Still images standardised to 128 × 128 × 1 pixels	RGB to greyscale, uint8 to float32 conversion, horizontal flipping for augmentation	TensorFlow and Keras	Attention mechanism (not visualized); no clinical validation; explanation quality not assessed
Hong et al. [84]	Classification	Convolutional Neural Network using EfficientNet-B0	Transfer learning with ImageNet pre-trained weights	Multi-label (normal, B-lines, consolidation, pleural effusion)	7580 images from 1645 patients	Deep learning CNN using EfficientNet-B0	Still images resized to 224 × 224 pixels, RGB channels	DICOM pseudonymisation, conversion to PNG, normalisation for ImageNet weights	Custom model (code available at GitHub)	Grad-CAM (minimal analysis); no clinical validation; explanation quality not assessed
Chaudhary et al. [85]	Classification	Convolutional Neural Network	Frame-level CNN with clip-level prediction algorithms (average, contiguous, majority vote)	Binary with adaptable thresholds for clinical scenarios	668 patients, 1425 clips, 266,670 frames	Deep learning CNN	Video clips deconstructed into frames	Frame extraction, masking, contrast adjustment (±20%), grid distortion (up to 25%)	Pleff-Net (PLeuralEFFusion neural NETwork)	Grad-CAM + temporal confidence plotting; visual inspection only; explanation quality not assessed

**Table 3:** Diagnostic accuracy measures. Diagnostic performance metrics of AI-based pleural effusion detection across the included studies, including sensitivity, specificity, accuracy, AUC, positive predictive value, and additional reported performance indicators. AUC = area under the curve, PPV = positive predictive value, CI = confidence interval, F1-score = harmonic mean of precision and recall, Sens = sensitivity, Spec = specificity, Acc = accuracy, Multi-class = classification with more than two categories, Binary = classification with two categories.

Study	Primary Outcome	Model Version	Sensitivity (%)	Specificity (%)	Accuracy (%)	AUC	PPV (%)	Additional Performance Metrics
Tsai et al. [81]	Pleural effusion detection	Video-based approach	Not reported individually	Not reported individually	91.12% (mean), 95.68% (best fold)	Not reported	Not reported	F1-score: 87.71% (best), 40.02% (worst); Precision: 87.29% (best), 38.85% (worst); Recall: 88.14% (best), 41.26% (worst)
Tsai et al. [81]	Pleural effusion detection	Frame-based approach	Not reported individually	Not reported individually	92.38% (mean), 96.75% (best fold)	Not reported	Not reported	F1-score: 90.47% (best), 34.98% (worst); Precision: 92.76% (best), 42.82% (worst); Recall: 88.28% (best), 29.57% (worst)
Chen et al. [82]	Multi-class classification (Class W—pleural effusion)	ResNet with Split-Attention	96.39%	100.00%	96.39%	99.80%	100.00%	Overall multi-class: 98.27% sensitivity, 99.41% specificity, 98.20% accuracy, 99.76% macro AUC
Huang et al. [83]	Pleural effusion detection and segmentation	Attention U-net	97% (range 91–100%)	91% (range 84–98%)	94% (range 91–98%)	0.98 (range 0.95–1.0)	93% (range 89–99%)	F1-score: 95% (range 92–98%); Dice coefficient: 0.86 (range 0.83–0.90)
Huang et al. [83]	Pleural effusion detection and segmentation	U-net	97% (range 93–100%)	86% (range 77–94%)	92% (range 90–95%)	0.97 (range 0.96–1.0)	90% (range 85–95%)	F1-score: 93% (range 91–96%); Dice coefficient: 0.82 (range 0.79–0.86)
Hong et al. [84]	Multi-label classification (temporal test)	EfficientNet-B0	82.3%	87.9%	86.2%	0.94 (95% CI: 0.93–0.95)	Not reported	Internal test: 86.9% sensitivity, 85.6% specificity; External test 1: 85.5% sensitivity, 76.5% specificity; External test 2: 70.6% sensitivity, 86.1% specificity
Chaudhary et al. [85]	Binary classification (holdout set)	General model	90.3%(95% CI: 83.0–94.6%)	89.0% (95% CI: 82.6–93.2%)	89.5% (95% CI: 85.0–92.8%)	93.9% (95% CI: 93.7–94.2%)	Not reported	Large effusion model: 95.5% sensitivity; Trauma model: 98.0% sensitivity, 67.0% specificity

**Table 4:** QUADAS-2 assessment of risk of bias and applicability for included studies. Assessment performed using the QUADAS-2 tool. Patient Selection evaluates risk of bias from study population and enrolment; Index Test evaluates risk of bias in the conduct or interpretation of the AI algorithm; Reference Standard assesses the risk of bias in the comparator (human LUS interpretation ± CT); Flow & Timing evaluates bias related to inclusion of patients and timing of tests. Applicability domains consider the relevance of each domain to the systematic review question. H: High risk; L: Low risk; QUADAS-2: Quality Assessment of Diagnostic Accuracy Studies, revised version. Risk of bias was independently evaluated by two reviewers, with disagreements resolved by consensus or by a third reviewer.

Study/Year	Patient Selection	Index Test	Reference Standard	Flow & Timing	Patient Selection Applicability	Index Test Applicability	Reference Standard Applicability
Tsai et al./2021 [81]	H	H	L	L	L	L	L
Chen et al./2023 [82]	H	H	L	L	L	L	L
Huang et al./2024 [83]	H	H	L	L	L	L	L
Hong et al./2024 [84]	H	H	L	L	L	L	L
Chaudhary et al./2025 [85]	H	H	L	L	L	L	L

## Data Availability

No new data were created or analyzed in this study.

## Supplementary Materials

The following supporting information can be downloaded at: https://www.mdpi.com/article/10.3390/diagnostics16010147/s1, Supplementary Material—Full search strategy; Supplementary Table S1—PRISMA 2020 Checklist; Supplementary Material—COI Diagnostics.

## Abbreviations

AI	Artificial intelligence
AG	Attention gate
AUC	Area under the curve
B0	Baseline model variant of EfficientNet
B-lines	Vertical artefacts in lung ultrasound
BLUE	Bedside Lung Ultrasound in Emergency
CI	Confidence interval (defined in tables, standard but explicitly included)
CNN	Convolutional neural network
CT	Computed tomography
DICOM	Digital Imaging and Communications in Medicine
DL	Deep learning
F1-score	Harmonic mean of precision and recall
GRADE	Grading of Recommendations, Assessment, Development and Evaluations
ICMJE	International Committee of Medical Journal Editors
ICU	Intensive care unit
IPD	Individual participant data
LUS	Lung ultrasound
ML	Machine learning
NPV	Negative predictive value
PACS	Picture Archiving and Communication System
PE	Pleural effusion
Pleff-Net	PLeural EFFusion neural NETwork
PNG	Portable Network Graphics format
POCUS	Point-of-care ultrasound
PPV	Positive predictive value
PRISMA	Preferred Reporting Items for Systematic Reviews and Meta-Analyses
PROSPERO	International Prospective Register of Systematic Reviews
QUADAS-2	Quality Assessment of Diagnostic Accuracy Studies 2
Reg-STN	Regularised Spatial Transformer Network
ResNet	Residual neural network
RGB	Red–green–blue colour model
TUS	Thoracic ultrasound
U-net	U-shaped convolutional network architecture
