# Supplementary material for: Artificial Intelligence-Based Automated Analysis for Pleural Effusion Detection on Thoracic Ultrasound: A Systematic Review
Source: Diagnostics (Basel). 2026 Jan 2;16(1):147. doi: 10.3390/diagnostics16010147 (PMC12785731; doi:10.3390/diagnostics16010147)
Supplement: Supplementary file 1 [file diagnostics-16-00147-s001.zip › Supplementary Material-Full search strategy.pdf]

Supplementary material - Full search strategy

Search strategy for MEDLINE (via PubMed)

Database: PubMed/MEDLINE

Date searched: August 20, 2025

Language restrictions: English

Search Components:

#1 Thoracic Ultrasound Terms:

("Ultrasonography"[MeSH] OR "Ultrasonography, Prenatal"[MeSH] OR ultrasound[tiab] OR ultrasonography[tiab] OR sonography[tiab] OR sonographic[tiab] OR "lung ultrasound"[tiab] OR LUS[tiab] OR "thoracic ultrasound"[tiab] OR "chest ultrasound"[tiab] OR "pulmonary ultrasound"[tiab] OR "pleural ultrasound"[tiab] OR POCUS[tiab] OR "point-of-care ultrasound"[tiab])

#2 Pleural Effusion Terms:

("Pleural Effusion"[MeSH] OR "pleural effusion"[tiab] OR "pleural fluid"[tiab])

#3 Artificial Intelligence Terms:

("Artificial Intelligence"[MeSH] OR "Machine Learning"[MeSH] OR "Deep Learning"[MeSH] OR "Algorithms"[MeSH] OR "Neural Networks, Computer"[MeSH] OR "Pattern Recognition, Automated"[MeSH] OR "Computer-Assisted Diagnosis"[MeSH] OR "artificial intelligence"[tiab] OR AI[tiab] OR "machine learning"[tiab] OR "deep learning"[tiab] OR "convolutional neural network*"[tiab] OR CNN[tiab] OR "computer vision"[tiab] OR algorithm*[tiab] OR automated[tiab] OR "image analysis"[tiab] OR "automatic detection"[tiab])

Final Search: #1 AND #2 AND #3

Results: 69 records

Search strategy for Scopus

Database: Scopus

Date searched: August 20, 2025

Language restrictions: English

Search Strategy:

(ultrasound OR ultrasonography OR sonography OR "lung ultrasound" OR "thoracic ultrasound" OR "chest ultrasound" OR "pleural ultrasound" OR pocus OR "point-of-care ultrasound") AND ("pleural effusion" OR "pleural fluid") AND ("artificial intelligence" OR ai OR "machine learning" OR "deep learning" OR "neural network" OR "neural networks" OR algorithm OR algorithms OR automated OR "computer vision" OR "image analysis" OR "automatic detection")

Search fields: Title, Abstract, Keywords

Results: 233 records

Search strategy for Cochrane Library

Database: Cochrane Central Register of Controlled Trials (CENTRAL)

Date searched: August 20, 2025

Language restrictions: English

Search Strategy:

(ultrasound OR ultrasonography OR sonography OR "lung ultrasound" OR "thoracic ultrasound" OR "chest ultrasound" OR "pleural ultrasound" OR POCUS) AND ("pleural effusion" OR "pleural fluid") AND ("artificial intelligence" OR AI

OR "machine learning" OR "deep learning" OR "neural network" OR algorithm OR automated OR "computer vision" OR "image analysis")
Search fields: Title, Abstract, Keywords
Results: 6 records

Search strategy for ClinicalTrials.gov
Database: ClinicalTrials.gov
Date searched: August 20, 2025
Language restrictions: English
Search Strategy:
AREA[ConditionSearch] pleural effusion AND AREA[InterventionSearch] (ultrasound OR ultrasonography OR "thoracic ultrasound" OR "lung ultrasound" OR POCUS) AND AREA[InterventionSearch] ("artificial intelligence" OR "machine learning" OR "deep learning" OR automated OR algorithm OR AI)
Search fields: Condition, Intervention
Study status: All studies
Results: 1 record

Search strategy for IEEE Xplore
Database: IEEE Xplore Digital Library
Date searched: August 20, 2025
Language restrictions: English
Search Strategy:
("thoracic ultrasound" OR "pleural ultrasound" OR "lung ultrasound" OR "chest ultrasound" OR POCUS) AND ("pleural effusion" OR "pleural fluid") AND ("artificial intelligence" OR "machine learning" OR "deep learning" OR "neural network" OR "computer vision" OR "image analysis" OR algorithm)
Search fields: Document Title, Abstract, Author Keywords, IEEE Keywords
Results: 11 records

Search strategy for Google Scholar
Database: Google Scholar
Date searched: August 20, 2025
Language restrictions: English
Search Strategy:
("thoracic ultrasound" OR "lung ultrasound") AND "pleural effusion" AND ("artificial intelligence" OR "machine learning")
Approach: First 100 results screened by relevance ranking.
