# Supplementary material for: Artificial Intelligence-Based Automated Analysis for Pleural Effusion Detection on Thoracic Ultrasound: A Systematic Review
Source: Diagnostics (Basel). 2026 Jan 2;16(1):147. doi: 10.3390/diagnostics16010147 (PMC12785731; doi:10.3390/diagnostics16010147)
Supplement: Supplementary file 1 [file diagnostics-16-00147-s001.zip › Supplementary Table S1-PRISMA 2020 Checklist.pdf]

PRISMA 2020 Checklist - Artificial intelligence-based automated analysis for pleural effusion detection on thoracic ultrasound: a systematic review

Section and Topic	Item #	Checklist item	Location where item is reported
**TITLE**			
Title	1	Identify the report as a systematic review.	Title page: "systematic review" is explicitly stated in the title
**ABSTRACT**			
Abstract	2	See the PRISMA 2020 for Abstracts checklist.	Abstract section: structured abstract with Background, Methods, Results, and Conclusions
**INTRODUCTION**			
Rationale	3	Describe the rationale for the review in the context of existing knowledge.	Introduction, paragraphs 1-7: discusses pleural effusion prevalence, diagnostic challenges, and AI potential
Objectives	4	Provide an explicit statement of the objective(s) or question(s) the review addresses.	Introduction, final paragraph: "This review therefore seeks to critically appraise studies evaluating AI-based automated analysis..."
**METHODS**			
Eligibility criteria	5	Specify the inclusion and exclusion criteria for the review and how studies were grouped for the syntheses.	Methods - Eligibility criteria section: separate subsections for Population, Index Test, Reference standard, Outcomes, and Eligible studies
Information sources	6	Specify all databases, registers, websites, organisations, reference lists and other sources searched or consulted to identify studies. Specify the date when each source was last searched or consulted.	Methods - Information sources and search strategy: "MEDLINE (via PubMed), Scopus, Google Scholar, IEEE Xplore, Cochrane CENTRAL, and ClinicalTrials.gov"; searched through 20 August 2025

Section and Topic	Item #	Checklist item	Location where item is reported
Search strategy	7	Present the full search strategies for all databases, registers and websites, including any filters and limits used.	Methods - Information sources and search strategy: states "complete search strings are provided in the Supplementary Material"
Selection process	8	Specify the methods used to decide whether a study met the inclusion criteria of the review, including how many reviewers screened each record and each report retrieved, whether they worked independently, and if applicable, details of automation tools used in the process.	Methods - Study selection and screening: "two reviewers independently screened...using Rayyan.ai software"; "Discrepancies were resolved by discussion or consultation with a third reviewer"
Data collection process	9	Specify the methods used to collect data from reports, including how many reviewers collected data from each report, whether they worked independently, any processes for obtaining or confirming data from study investigators, and if applicable, details of automation tools used in the process.	Methods - Data extraction: "Data were extracted independently by at least two reviewers using pre-specified forms, with disagreements resolved through discussion or adjudication by a third reviewer"
Data items	10a	List and define all outcomes for which data were sought. Specify whether all results that were compatible with each outcome domain in each study were sought (e.g. for all measures, time points, analyses), and if not, the methods used to decide which results to collect.	Methods - Eligibility criteria - Outcomes: Primary outcomes (sensitivity, specificity, PPV, NPV, accuracy, AUC) and Secondary outcomes listed
	10b	List and define all other variables for which data were sought (e.g. participant and intervention characteristics, funding sources). Describe any assumptions made about any missing or unclear information.	Methods - Data extraction: bullet points list study characteristics, patient characteristics, ultrasound specifications, AI

Section and Topic	Item #	Checklist item	Location where item is reported
			system details, reference standard, and subgroup analyses
Study risk of bias assessment	11	Specify the methods used to assess risk of bias in the included studies, including details of the tool(s) used, how many reviewers assessed each study and whether they worked independently, and if applicable, details of automation tools used in the process.	Methods - Risk of bias and quality assessment: "Risk of bias was evaluated using QUADAS-2"; "Assessments were conducted independently by two reviewers"
Effect measures	12	Specify for each outcome the effect measure(s) (e.g. risk ratio, mean difference) used in the synthesis or presentation of results.	Methods - Eligibility criteria - Outcomes: lists sensitivity, specificity, PPV, NPV, accuracy, and AUC
Synthesis methods	13a	Describe the processes used to decide which studies were eligible for each synthesis (e.g. tabulating the study intervention characteristics and comparing against the planned groups for each synthesis (item #5)).	Methods - Review design and Data synthesis: explains heterogeneity precluded meta-analysis; structured narrative synthesis employed
	13b	Describe any methods required to prepare the data for presentation or synthesis, such as handling of missing summary statistics, or data conversions.	Methods - Data extraction: describes extraction of performance metrics and study characteristics; notes IPD were not sought
	13c	Describe any methods used to tabulate or visually display results of individual studies and syntheses.	Methods - Data synthesis: "structured narrative synthesis integrated with four tables"; Results mentions Tables 1-3 and Figure 1
	13d	Describe any methods used to synthesize results and provide a rationale for the choice(s). If meta-analysis was performed, describe the model(s), method(s) to identify the presence and extent of statistical heterogeneity, and software package(s) used.	Methods - Data synthesis: "structured narrative synthesis, complemented by tabular summaries";

Section and Topic	Item #	Checklist item	Location where item is reported
			explains why meta-analysis was not performed due to heterogeneity
	13e	Describe any methods used to explore possible causes of heterogeneity among study results (e.g. subgroup analysis, meta-regression).	Results section: subgroup analyses by effusion characteristics and patient populations are described
	13f	Describe any sensitivity analyses conducted to assess robustness of the synthesized results.	Not applicable. Due to substantial heterogeneity precluding meta-analysis, sensitivity analyses were not performed. Robustness was instead assessed through subgroup analyses by effusion characteristics and patient populations (see Results - Performance by effusion characteristics)
Reporting bias assessment	14	Describe any methods used to assess risk of bias due to missing results in a synthesis (arising from reporting biases).	Methods - Risk of bias and quality assessment: "Potential bias due to missing or unpublished results was considered and discussed qualitatively"
Certainty assessment	15	Describe any methods used to assess certainty (or confidence) in the body of evidence for an outcome.	Methods - Certainty of evidence: "evaluated in accordance with the GRADE framework"; describes five domains assessed
**RESULTS**			
Study selection	16a	Describe the results of the search and selection process, from the number of records identified in the search to the number of studies	Results, first

Section and Topic	Item #	Checklist item	Location where item is reported
		included in the review, ideally using a flow diagram.	paragraph: "five studies were identified which met the eligibility criteria, as illustrated in the PRISMA flow diagram (Figure 1)"
	16b	Cite studies that might appear to meet the inclusion criteria, but which were excluded, and explain why they were excluded.	Figure 1 (PRISMA flow diagram) reports the number of excluded studies with categories of exclusion reasons at full-text screening stage.
Study characteristics	17	Cite each included study and present its characteristics.	Results - Study characteristics and settings: references 50-54 cited; Table 1 presents detailed characteristics
Risk of bias in studies	18	Present assessments of risk of bias for each included study.	Results - Risk of bias section: detailed assessment presented; Table 4 summarizes QUADAS-2 assessments
Results of individual studies	19	For all outcomes, present, for each study: (a) summary statistics for each group (where appropriate) and (b) an effect estimate and its precision (e.g. confidence/credible interval), ideally using structured tables or plots.	Results - Diagnostic performance metrics: detailed performance data for all studies; Table 3 presents comprehensive diagnostic accuracy measures
Results of syntheses	20a	For each synthesis, briefly summarise the characteristics and risk of bias among contributing studies.	Results - multiple subsections describe study characteristics; Risk of bias section summarizes QUADAS-2 findings
	20b	Present results of all statistical syntheses conducted. If meta-analysis was done, present for each the summary estimate and its precision (e.g. confidence/credible interval) and measures of statistical heterogeneity. If comparing groups, describe the direction of the effect.	Results - Diagnostic performance metrics and Statistical

Section and Topic	Item #	Checklist item	Location where item is reported
			significance sections: performance ranges reported (sensitivity 70.6-100%, specificity 67-100%, AUC 0.77-0.998)
	20c	Present results of all investigations of possible causes of heterogeneity among study results.	Results - Performance by effusion characteristics: subgroup analyses by effusion size and patient population
	20d	Present results of all sensitivity analyses conducted to assess the robustness of the synthesized results.	Not applicable. Sensitivity analyses were not performed as narrative synthesis was used instead of meta-analysis due to substantial heterogeneity. Robustness of findings was assessed through subgroup analyses by effusion characteristics and patient populations (Results - Performance by effusion characteristics section)
Reporting biases	21	Present assessments of risk of bias due to missing results (arising from reporting biases) for each synthesis assessed.	Results - Certainty of evidence (GRADE): Publication Bias subsection discusses limited assessment due to small study number
Certainty of evidence	22	Present assessments of certainty (or confidence) in the body of evidence for each outcome assessed.	Results - Certainty of evidence (GRADE): comprehensive GRADE assessment with overall certainty rated as "moderate

Section and Topic	Item #	Checklist item	Location where item is reported
			for sensitivity and specificity"
**DISCUSSION**			
Discussion	23a	Provide a general interpretation of the results in the context of other evidence.	Discussion - Interpretation of findings: discusses findings in context of technical feasibility and clinical application
	23b	Discuss any limitations of the evidence included in the review.	Discussion - Study limitations and methodological considerations: extensive discussion of methodological heterogeneity, validation issues, and evidence gaps
	23c	Discuss any limitations of the review processes used.	Discussion - Study limitations section: mentions EMBASE not accessed, discusses QUADAS-2 limitations for AI assessment
	23d	Discuss implications of the results for practice, policy, and future research.	Discussion - Implications for clinical practice and Research gaps and future directions: comprehensive sections on both topics
**OTHER INFORMATION**			
Registration and protocol	24a	Provide registration information for the review, including register name and registration number, or state that the review was not registered.	Methods - Review design: "registered with PROSPERO (CRD420251128416)”
	24b	Indicate where the review protocol can be accessed, or state that a protocol was not prepared.	Methods - Review design: PROSPERO registration number provided (protocol accessible via PROSPERO

Section and Topic	Item #	Checklist item	Location where item is reported
			database)
	24c	Describe and explain any amendments to information provided at registration or in the protocol.	No amendments were made to the protocol after registration.
Support	25	Describe sources of financial or non-financial support for the review, and the role of the funders or sponsors in the review.	No funding was received for this systematic review. The review was conducted independently by the authors without financial or non-financial support from external sources
Competing interests	26	Declare any competing interests of review authors.	The authors declare that they have no known competing financial interests or personal relationships
Availability of data, code and other materials	27	Report which of the following are publicly available and where they can be found: template data collection forms; data extracted from included studies; data used for all analyses; analytic code; any other materials used in the review.	Full search strategies are available in Supplementary Material. Data extraction forms and extracted data are available from the corresponding author upon reasonable request

From: Page MJ, McKenzie JE, Bossuyt PM, Boutron I, Hoffmann TC, Mulrow CD, et al. The PRISMA 2020 statement: an updated guideline for reporting systematic reviews. BMJ 2021;372:n71. doi: 10.1136/bmj.n71. This work is licensed under CC BY 4.0. To view a copy of this license, visit https://creativecommons.org/licenses/by/4.0/
